# Mechanisms underlying the role of TNF-α and IL-1β preconditioned exosomes derived from human umbilical cord mesenchymal stem cells in wound healing

**DOI:** 10.3389/fimmu.2026.1713004

**Published:** 2026-02-17

**Authors:** Ziyue Zhou, Rui Cao, Lei Shi, Wenhu Jin

**Affiliations:** 1 Department of Burns and Plastic Surgery, Affiliated Hospital of Zunyi Medical University, Zunyi, Guizhou, China; 2 The 2011 Collaborative Innovation Center of Tissue Damage Repair and Regeneration Medicine, Affiliated Hospital of Zunyi Medical University, Zunyi, Guizhou, China; 3 The Collaborative Innovation Center of Tissue Damage Repair and Regeneration Medicine of Zunyi Medical University, Zunyi, Guizhou, China

**Keywords:** exosomes, exosomes sequence, HUC-MSCs, inflammation, wound healing

## Abstract

**Objective:**

In this study, human umbilical cord mesenchymal stem cells (hUC-MSCs) were stimulated with tumor necrosis factor-α (TNF-α) and interleukin-1β (IL-1β) to obtain preconditioned exosomes (Exos). Comparative miRNA profiling was performed between cytokine-primed Exos and conventionally cultured counterparts to identify differentially expressed miRNAs. Functional validation of candidate miRNAs will elucidate their mechanistic roles in promoting cutaneous repair, thereby advancing the clinical translation of Exos-based regenerative therapies.

**Methods:**

We successfully extracted and characterized hUC-MSCs derived Exos (hUCMSCs-Exos) and classified them into Exos obtained under normal culture conditions (Con-Exos) and those stimulated by TNF-α+IL-1β obtained Exos (TNF-α+IL-1β-Exos). The wound healing rate was observed and counted by establishing a mouse whole skin defect wound model *in vivo*. *In vitro*, human umbilical vein endothelial cells (HUVECs) were stimulated with two groups of hUCMSCs-Exos to observe the tube formation of HUVECs and the results of 5-ethynyl-2’-deoxyuridine (EdU) assay.Then, the differential miRNAs in the two groups of hUCMSCs-Exos were detected and validated to identify the candidate effector miRNAs, which were then analyzed by databases and searched and screened for common target genes.

**Results:**

TNF-α+IL-1β-Exos accelerated wound healing more than Con-Exos, mainly by increasing collagen deposition and expression of the angiogenic marker CD31. Sequencing and bioinformatics analyses revealed that the key miRNA for both co-actions was miR-215-5p. Cellular experiments showed that miR-215-5p mimics promoted HUVECs tubulogenesis and proliferation, whereas the inhibitor effect was not significant. In animal experiments, miR-215-5p mimics also significantly accelerated wound healing in mice.

**Conclusion:**

TNF-α+IL-1β-Exos demonstrated superior wound healing efficacy compared to Con-Exos. This enhanced therapeutic effect may be attributed to the elevated expression of miR-215-5p in TNF-α+IL-1β-Exos. Mechanistically, miR-215-5p activates the *WNK1*/*p-Smad3*/*VEGF-A* signaling axis, promoting angiogenesis and accelerating cutaneous wound repair.

## 1 Background

Wound healing is a complex biological process involving dynamic regulation across three phases: inflammatory, proliferative, and remodeling. This process relies critically on interactions between keratinocytes, endothelial cells, fibroblasts, and the extracellular matrix (1). While moderate inflammation helps clear damaged tissue and pathogens to establish the foundation for tissue repair, excessive or prolonged inflammatory responses may lead to chronic wounds and impaired healing (2).

MSCs have shown great promise in regenerative medicine due to their immunomodulatory and tissue regeneration capabilities. By interacting with immune cells and reparative cells in the wound microenvironment, MSCs can modulate inflammatory responses and promote tissue repair (3). For instance, they facilitate macrophage polarization toward the M2 phenotype to reduce inflammation and accelerate wound healing (4). Notably, MSCs functionality is highly microenvironment-dependent, with significant biological variations observed across different tissue sources or microenvironmental conditions (5).

Research indicates that MSCs primarily exert their effects through paracrine mechanisms (6). The cytokines, chemokines, growth factors, and Exos they secrete collectively regulate inflammation and tissue repair (7). Among these, Exos, 30–150 nm membrane vesicles have garnered particular attention as key paracrine mediators. Exos facilitate intercellular communication by carrying functional molecules like proteins and miRNAs, with their biological effects closely tied to miRNA enrichment profiles (8). Compared to MSCs, Exos offer several advantages: (1) Enhanced targeting specificity through membrane receptor recognition; (2) Superior stability for long-term storage (9); (3) Smaller size enabling penetration of physiological barriers (e.g., blood-brain or blood-testis barriers) (10); (4) Comparable or even superior reparative and immunomodulatory capabilities to their parent MSCs, which is the most critical.

However, Exos derived from conventionally cultured MSCs suffer from limitations such as low yield and insufficient functionality. To address this, researchers have explored pretreatment strategies, including cytokine stimulation, hypoxia, or genetic modification to enhance Exos performance. For instance, melatonin-pretreated MSCs-derived Exos promote diabetic wound healing by activating the *PTEN/AKT* pathway, while hypoxia-induced Exos exhibit enhanced cartilage repair capabilities (11, 12). Notably, when MSCs are pretreated with inflammatory cytokines (e.g., IFN-γ, TNF-α, and IL-1β) to mimic the inflammatory microenvironment *in vivo*, their derived Exos demonstrate significantly improved immunomodulatory properties (13, 14).

Based on existing research, we recognize that the complexity of the early inflammatory wound microenvironment cannot be fully replicated by a single factor. TNF-α and IL-1β, serving as core mediators of early wound inflammation, exhibit synergistic effects: TNF-α activates the *NF-κB* pathway to promote angiogenesis, while IL-1β stimulates fibroblast proliferation, providing matrix support for vascular formation. Their combined use offers a more comprehensive simulation of the wound microenvironment, thereby yielding Exos with superior functionality. Moreover, compared to single-factor pretreatment, dual-cytokine pretreatment generates Exos with higher efficacy in accelerating wound healing.

Therefore, this study employs TNF-α and IL-1β in combination to pretreat hUC-MSCs. The secreted Exos will be isolated and their miRNAs expression profiles analyzed to identify key effector miRNAs involved in wound healing regulation. By elucidating the underlying molecular mechanisms, we aim to provide a novel theoretical foundation and therapeutic strategy for the clinical translation of MSCs-Exos-based regenerative therapies.

## 2 Materials and methods

The main reagents and instruments used in the experiments are listed in **Tables 1**, **2**.

**Table 1:** Experimental instruments.

Instrument	Manufacturer
Benchtop Low-Speed Centrifuge	TD5A-WS, CHN
High-Speed Refrigerated Centrifuge	Beckman coulter, USA
Upright Fluorescence Microscope	Carl Zeiss, GER
Scanning Electron Microscope (SEM)	Olympus, VS200, JPN
Full-Wavelength Microplate Reader	Bio-Tek, USA
Real-Time Fluorescence Quantitative PCR	Thermo Fisher Scientific, USA
Gradient PCR Thermocycler	Thermo Fisher Scientific, USA
Ultra-Micro Spectrophotometer	Thermo Fisher Scientific, USA
Tissue Embedding Machine/Slicer/Dehydrator	Leica, GER
Animal Anesthesia Machine	RWD Life Science, CHN
Gen5 Microplate Reader	Bio-Tek, USA
TEM	Hitachi, JPN
NTA	NanoFCM, CHN
Laser Scanning Confocal Microscope	Leica, GER

**Table 2:** Experimental reagents.

Reagent	Manufacturer
Masson Staining Kit	Coolaber, CHN (Beijing)
IHC Staining Kit	Maixing (Fuzhou) Biotech Co., Ltd.
DAPI	Solarbio, CHN (Beijing)
BCA Protien Assay Kit	Solarbio, CHN (Beijing)
IgG H&L (HRP)	ABclonal, CHN (Wuhan)
Glycine	Solarbio, CHN (Beijing)
QuickBlock™ Western	Beyotime, CHN (Shanghai)
Citrate buffer(PH6)	Zhongshan Golden Bridge (Beijing) Biotechnology Co., Ltd.
PBS	Solarbio, CHN (Beijing)
miRNeasy Mini kit	Qiagen, 217004
RevertAid Synthesis Kit	Thermo Fisher Scientific, USA
SYBR Green qPCR Master	Roche, CH
FITC Mouse Anti-Human CD9	BD, USA
FITC Mouse Anti-Human CD63	BD, USA
FITC Mouse Anti-Human CD81	BD, USA
PKH26	Solarbio, CHN (Beijing)
BSA	Sigma, V900933-100G
Mounting Medium(DAPI)	Vector, CHN
ECM medium	SCLENCELL, CHN (Beijing)
Fetal Bovine Serum FBS	SCLENCELL, CHN (Beijing)
VEGF-A	Proteintech, CHN (Wuhan)
P-smad3	HuaBio, CHN (Hangzhou)
WNK1	HuaBio, CHN (Hangzhou)
CD31	Abcam, USA
CD206	HuaBio, CHN (Hangzhou)
iNOS	ABclonal, CHN
Lipofectamine 3000	Thermo Fisher Scientific, USA
miR-215-5p inhibitor	GenePharma (Shanghai) Co., Ltd.
miR-215-5p mimic	GenePharma (Shanghai) Co., Ltd.

### 2.1 Cell culture and identification

All human umbilical cord samples from healthy full-term neonates were provided by the Affiliated Hospital of Zunyi Medical University. The collection process strictly adhered to the informed consent principle (Ethics Approval No.: KLLY-2023-045). HUC-MSCs were isolated using the tissue explant method and cultured in mesenchymal stem cell specialized medium. The cells were maintained in a humidified incubator at 37°C with 5% CO_2_. Cell surface markers (CD105, CD29, CD44, CD45, CD34, HLA-DR) were characterized by flow cytometry. HUVECs were purchased from the Cell Bank of the Chinese Academy of Sciences (Shanghai) and cultured in DMEM or RPMI 1640 medium supplemented with 10% fetal bovine serum (FBS) under the same conditions (37°C, 5% CO_2_).

### 2.2 Exos isolation and characterization

After treating hUC-MSCs for 48 hours with either basal medium (control group) or medium supplemented with 20 ng/mL TNF-α and IL-1β (experimental group), the supernatant was collected. The cell supernatant was subjected to differential centrifugation to remove dead cells, debris, and large vesicles (4°C, 500 g, 10 min/4°C, 2000 g, 20 min/4°C, 10,000 g, 30 min). The supernatant was then transferred to a 100 kDa ultrafiltration tube and centrifuged at 2,500 g for 40 min at 4°C to collect the filtrate. The filtrate was transferred to an ultracentrifuge tube and centrifuged twice at 100,000 × g for 100 min each time. The final pellet was resuspended in 200 µL of sterile PBS and stored at -80°C.

The morphological of hUCMSCs-Exos were examined by transmission electron microscopy (TEM). Particle size distribution was analyzed by nanoparticle tracking analysis (NTA). Surface-specific exosomal markers (CD9, CD63, and CD81) were detected by flow cytometry.

### 2.3 Exos tracing experiments

50 µg of Exos were mixed with 2 µl of PKH26 dye in Dilution C (final volume: 500 µl) and incubated at room temperature for 5 min. The reaction was terminated by adding 1% BSA. After ultracentrifugation (120,000 g, 60 min, 4°C), the pellet was resuspended in 500 µL of DMEM. Cells were digested and inoculated into 24-well plates, followed by overnight culture. After PBS washing, the stained Exos were added and co-incubated for 24 h. Then fixed with 4% paraformaldehyde for 10 min. Finally, DAPI blocked for confocal microscopy.

### 2.4 Tube formation assay

125 µl of matrix gel was added to a 48-well plate and incubate at 37°C for 30 min. The cells were digested and centrifuged, resuspended in serum-free medium. Prepared a 500 µL cell-suspension containing 4×10^4^ cells in complete medium.Then seeded onto the Matrigel-coated wells and incubate at 37°C for 2 h before imaging.

### 2.5 EdU assay

Log-phase cells were digested and inoculated into 6-well plates and incubated at 37°C, 5% CO_2_ for 24 h to adhere. The cells were then labeled by incubating with 20 µM 2× EdU working solution (final concentration 10 µM) for 2 h. Permeabilization was performed using PBS containing 0.3% Triton X-100. The cell-covered slides were fixed with 4% paraformaldehyde and rinsed three times with PBS (5 min each). After treatment with permeabilization buffer, the EdU reaction cocktail was added and incubated at 37°C for 1 h protected from light. Then counterstained with DAPI for 15 min before mounting with antifade reagent. Imaging was performed using an inverted fluorescence microscope.

### 2.6 Immunofluorescence staining (TSA method)

The dewaxed samples were rehydrated and treated with citrate-based antigen retrieval buffer (pH 6.0), followed by permeabilization with a membrane-breaking solution for 10 min before washing. Then blocked with 10% goat serum for 30 min. First round of staining: primary antibody overnight at 4°C → PBS → HRP secondary antibody for 50 min → tyrosine salt-CY3 for 20 min → PBS → microwave repair. Second round of staining: closed for 10 min → secondary antibody overnight at 4°C → HRP secondary antibody for 50 min → tyramide-488 for 20 min → PBS. Third round of staining: triple antibody overnight at 4°C → HRP secondary antibody 50 min → tyramide-CY5–20 min → PBS. DAPI staining for 10 min. The captured images were acquired using a fluorescence microscope after mounting with antifade reagent.

### 2.7 Cell transfection experiments

When the cell confluence reached 50%-70%, the procedure was initiated by replacing the medium without serum 1 h before transfection. The miRNA mimics were diluted to a final concentration of 50–100 nM, then both the miRNA mimics and the transfection reagent were separately diluted in Opti-MEM (usually 1:1) incubating for 15–20 min. The resulting complexes were gently added dropwise to the cells at 37°C for 4–6 h, then changed into complete medium cultured for an additional 24–48 h. Transfection efficiency was subsequently verified by quantifying miRNA expression levels using qPCR.

### 2.8 Quantitative real-time polymerase chain reaction

Total RNA was extracted from exosomes or cells using TRIzol reagent. MiRNA First Strand cDNA Synthesis (Plus Tail) Kit was used. Reverse transcription of mRNA into cDNA was performed using the RevertAid First Strand cDNA Synthesis Kit, then mRNA expression levels were detected using the SYBR Green-based assay, and GAPDH and U6 levels were used as internal references. Real-time PCR (RT-qPCR) was performed on a QuantStudio 3 real-time PCR system using SYBR PCR master mix. Relative miRAN expression levels were analyzed using the 2-ΔΔCt method. Primer sequences are shown in **Table 3**.

**Table 3:** Experimental related primer sequences.

Primers	Forward	Reverse
miR-1-3p	TCGGCAGGTGGAATGTAAAGAAGT	CAGTGCAGGGTCCGAGGTAT
miR-215-5p	GCCGAGATGACCTATGAATTG	CAGTGCAGGGTCCGAGGTAT
miR-126-3p	TCGTACCGTGAGTAAT	CAGTGCAGGGTCCGAGGTAT
miR-146a-5p	TCGGCAGGTGAGAACTGAATTCCA	CAGTGCAGGGTCCGAGGTAT
miR-139-5p	TCTACAGTGCACGTGTC	CAGTGCAGGGTCCGAGGTAT
miR-133-3p	GCCGAGTTTGGTCCCCTTCAAC	CAGTGCAGGGTCCGAGGTAT
WNK1	AACAAGCCGTTGTAGGCTCG	GACCGTCATTGGACATTCCCA
GAPDH	TGGCCTTCCGTGTTCCTAC	GAGTTGCTGTTGAAGTCGCA
U6	GCACATATACGCTTCGGCATAAAAT	CATTTGCGGCTTCACGATGTCAT

### 2.9 Animal experiments

Male C57B/L6 mice (n=28) at 6–8 weeks and weighing 22–25 g were purchased from Skibbes Biotechnology Co., Ltd.After mice were anesthetized with 4-5% isoflurane, their backs were shaved and cleaned. A sterile biopsy punch was used to create full-thickness circular skin wounds measuring 1 cm in diameter on the dorsum. Mice were randomly assigned to each group (n=7) in the next two animal experiments. In the first animal experiment, The control group received 200 µL of HUCMSCs-Exos (Con-Exos, 1 µg/µL), while the experimental group received 200 µL of TNF-α+IL-1β-Exos (TNF-α+IL-1β-Exos, 1 µg/µL); In the second experiment, control and experimental animals were injected with 200 µL of PBS (NC) or miR-215-5p mimics (MIMICS), respectively. Subcutaneous multi-point injections were administered along the wound edges. The images of wound healing were recorded on postoperative days 0, 3, 5, 7, 9, and 11, analyzed by ImageJ software, and the wound healing efficiency was calculated using the following formula: Healing efficiency (%) = [(Initial wound area - Wound area on day x)]/Initial wound area] * 100%.

### 2.10 Histological analysis, immunohistochemistry, immunofluorescence staining

The collected skin tissues were fixed in 4% paraformaldehyde for 48 h, dehydrated and embedded in paraffin before cut into μm-thick sections. These sections underwent standard H&E staining and Masson’s trichrome staining, after which, the structure of the samples was observed under a microscope. The collagen volume fraction was measured using ImageJ software, and the average values were calculated to assess the trends in collagen content changes.

The tissue sections were placed in a citrate repair buffer at pH 6 and boiled for antigen retrieval. Then, 3% hydrogen peroxide was applied to block endogenous peroxidase activity, followed by a 15-min incubation in the dark. After using non-specific blocking buffer at 37°C for 1 h, the primary antibody was added and incubated overnight at 4°C. The secondary antibody was then applied and incubated at room temperature for 45 min. DAB substrate was used for color development under microscopic observation. Finally, the sections were counterstained with hematoxylin.

The antigen retrieval process in immunofluorescence staining was similar to that of IHC. The endogenous peroxidase was blocked by applying 3% hydrogen peroxide, followed by incubation in the dark for 15 min. Then, treated with 5% BSA blocking buffer at 37°C for 30 min. The prepared primary antibody was applied, and the slides were incubated overnight at 4°C in a humidified chamber. A fluorescently labeled secondary antibody was added and incubated at room temperature for 45 min, followed by DAPI staining. Finally, samples were observed and photographed under an upright fluorescence microscope.

### 2.11 Western blot

The electrophoresis gel was prepared according to the SDS-PAGE kit instructions, and the protein sample for 95°C 5 min was cooled to 4°C for later use. Protein samples, protein Maker were added to the electrophoresis gel lane. After the power supply is turned on, the concentrated gel voltage is adjusted to 80 V. After the bromopholl blue band enters the lower gel to 120 V, the electrophoresis is continued until the bromopholl blue band reaches the bottom of the lower gel. Methanol activated the PVDF membrane for 30s, and the membrane transfer time was determined according to the protein molecular weight. The membrane was transferred at 5% BSA TBST and blocked with a decolorization shaker at room temperature. The decolorization shaker was incubated with primary antibody for 4°C 14 h. 20% Tween was washed 10 min 3 times. The corresponding secondary antibody was added, and the decolorization shaker was incubated for 1 h at room temperature. The ECL developer was added dropwise, protected from light for 10s before exposure using a chemiluminescence instrument and photographed for preservation.

### 2.12 Statistical analysis

Statistical analyses were performed with GraphPad Prism 8.0 software (GraphPad Software Inc., USA). Data are presented as mean ± standard deviation (SD). Differences between groups were determined by independent samples t-tests, one-way analysis of variance (ANOVA) was applied in the comparative analysis of data from multiple groups. Statistical significance was set at * P < 0.05, ** P < 0.01, *** P <0.001, **** P < 0.0001.

## 3 Results

### 3.1 Extraction and identification of hUC-MSCs

**Figure 1:**
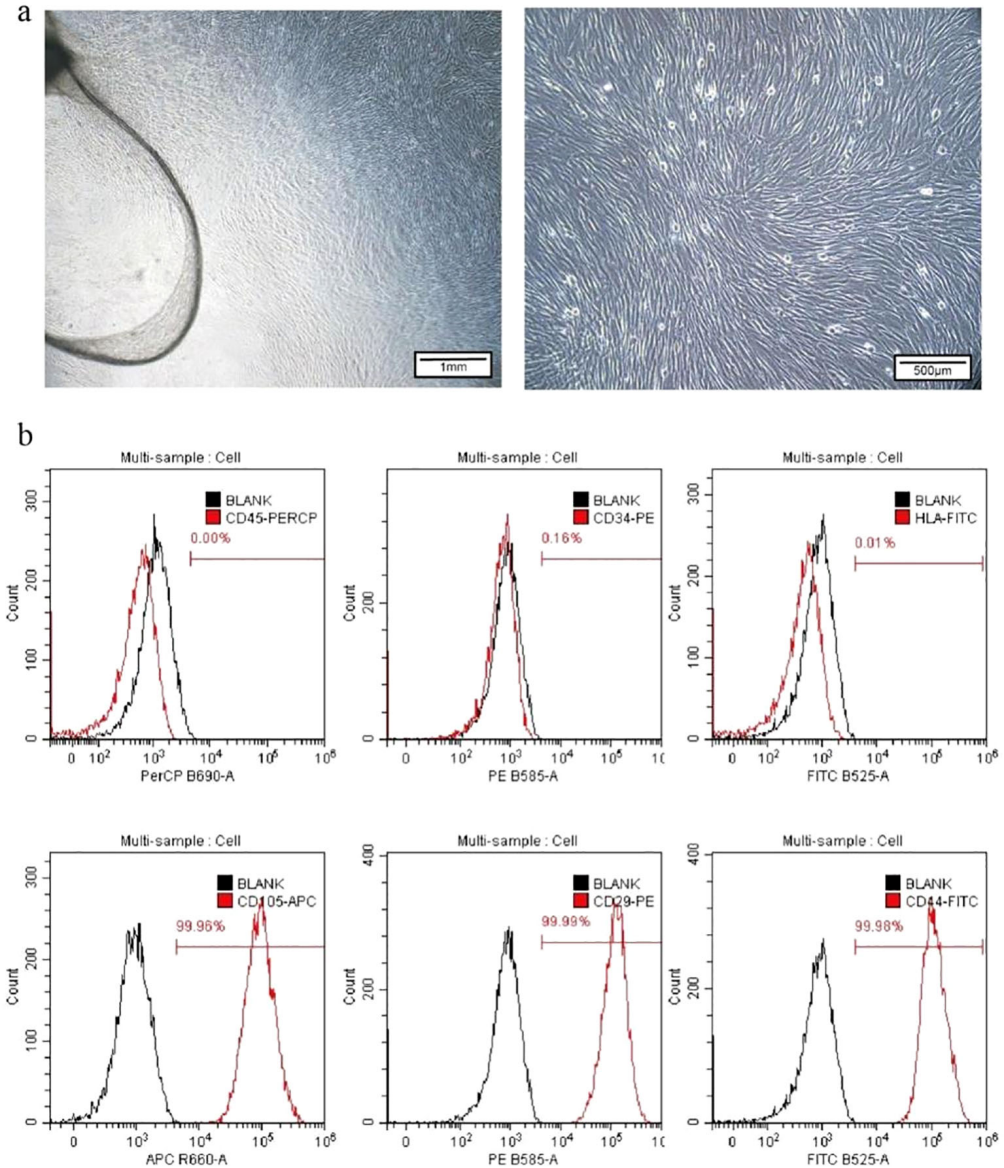
Extraction and identification of hUC-MSCs. **(a)** Microscopic morphology of hUC-MSCs (left: 20×, scale bar = 1000 µm; right: 40×, scale bar = 500 µm). **(b)** Flow cytometry analysis of hUC-MSCs for surface markers (CD45, CD34, HLA-DR, CD105, CD29, and CD44).

hUC-MSCs were successfully isolated from the umbilical cord tissue of full-term cesarean-delivered newborns using the tissue explant method. Observation under an inverted phase-contrast microscope revealed that these cells exhibited a uniform spindle-shaped morphology and demonstrated typical adherent-dependent growth patterns (**Figure 1A**, left). At 40× magnification, the cells were arranged in a fish school-like or whirlpool-like pattern (**Figure 1A**, right). Further analysis by flow cytometry to detect surface markers showed that the positive expression rates of CD45, CD34, and HLA-DR were 0.00%, 0.16%, and 0.01%, respectively, while the positive rates for CD105, CD29, and CD44 were 99.96%, 99.99%, and 99.98%, respectively (**Figure 1B**). These results confirmed that the cells extracted via the tissue explant method were hUC-MSCs.

### 3.2 Isolation and characterization of Con-Exos and TNF-α+IL-1β-Exos

**Figure 2:**
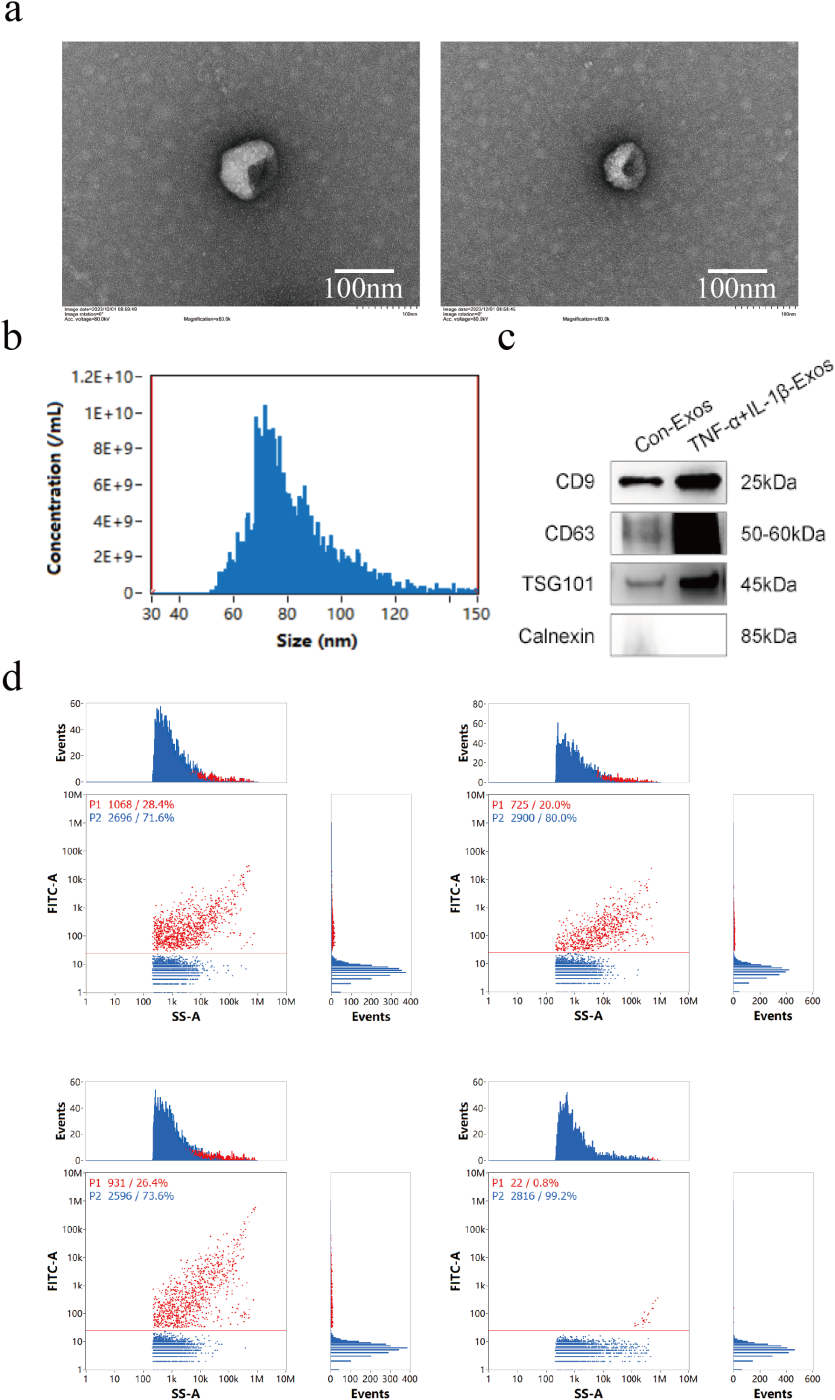
Isolation and characterization of Con-Exos and TNF-α+IL-1β-Exos. **(a)** Morphology of Exos under TEM (scale bar = 100 nm). **(b)** NTA analysis revealed a diameter range of 30–150 nm for Exos. **(c)** WB analysis of exosomal markers (CD9, CD63, TSG101, Calnexin). **(d)** Flow cytometry analysis of exosomal markers (CD9, CD63, and CD81).

Exos were isolated and purified from the culture supernatant of hUC-MSCs using differential centrifugation and ultracentrifugation. TEM analysis revealed that the obtained vesicles exhibited the characteristic cup-shaped morphology, which intact membrane structures consistent with the typical morphological features of Exos (**Figure 2A**). NTA analysis demonstrated that the vesicle diameter ranged from 30 to 150 nm, with an average size of 82.4 nm, which aligned with the known size distribution of Exos (**Figure 2B**). Western blot analysis confirmed positive expression of vesicle surface-specific markers CD9, CD63, and TSG101, while the negative protein Calnexin was not detected (**Figure 2C**). Furthermore, nanoflow cytometry detection of vesicle surface-specific markers showed positive rates of 28.4% for CD9, 26.4% for CD63, and 20% for CD81 (**Figure 2D**). These results indicated that the successful isolation of Exos.

### 3.3 TNF-α+IL-1β-Exos accelerate wound healing by promoting angiogenesis and collagen deposition

**Figure 3:**
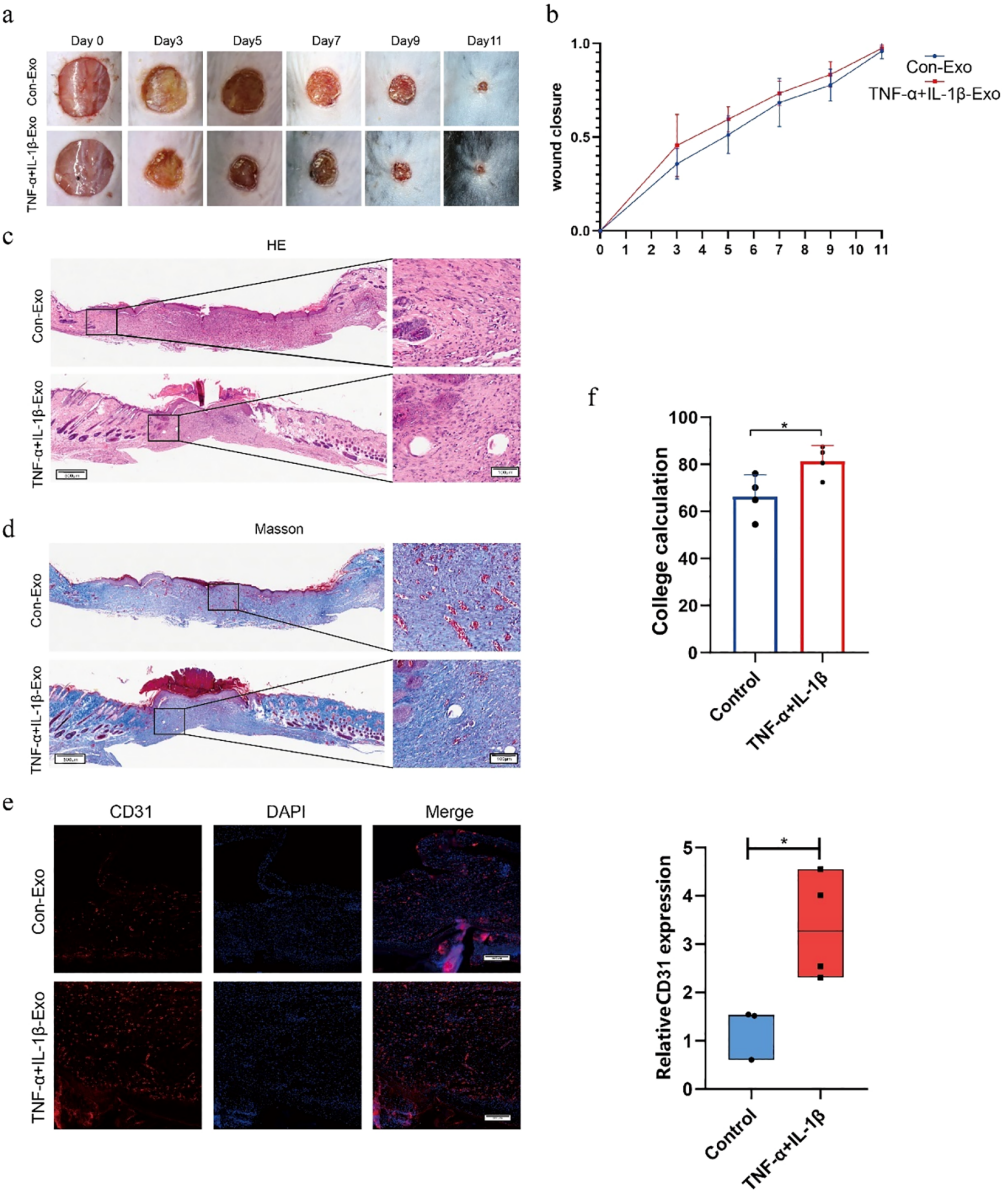
TNF-α+IL-1β-Exos accelerate wound healing by promoting angiogenesis and collagen deposition. **(a)** Photographs of wound healing in mice on days 0, 3, 5, 7, 9, and 11 (scale bar = 1 cm). **(b)** Statistical analysis of wound healing rates in mice across different time points (n = 7). **(c)** H&E staining of wound tissues on day 11 (200×, scale bar = 100 µm). **(d)** Masson’s trichrome staining of wound tissues on day 11 (200×, scale bar = 100 µm). **(e)** CD31 immunofluorescence staining of wound tissues on day 11 (100×, scale bar = 200 µm). **(f)** Statistical analysis of collagen area and CD31^+^ expression (*P < 0.05, n = 3).

To evaluate the impact of Exos on skin repair, a C57BL/6 mouse model was established by creating full-thickness skin wounds with a diameter of 1 cm on the dorsal region. The experimental groups were divided into a control group (Con-Exos) and a cytokine-stimulated group (TNF-α+IL-1β-Exos), with Exos administered via multi-point injections around the wound edges. The wound healing process was monitored over an 11-day period, and photographic documentation was performed on days 0, 3, 5, 7, 9, and 11. The percentage of wound closure was calculated using Image J software to assess the repair efficacy across groups. The results demonstrated that compared to the Con-Exos group, the TNF-α+IL-1β-Exos group significantly accelerated wound healing, with the most pronounced effects observed on days 3 and 5 (**Figures 3A, B**). This finding was further corroborated by H&E staining of wound tissues collected on day 11 (**Figure 3C**). Additionally, Masson’s trichrome staining and CD31 immunofluorescence analysis revealed that the TNF-α+IL-1β-Exos group exhibited enhanced collagen deposition and a marked increase in the number of newly formed capillaries compared to the control group (* P < 0.05) (**Figures 3D–F**). These findings suggest that the early-stage acceleration of wound healing by TNF-α+IL-1β-Exos may be closely associated with the promotion of collagen deposition and angiogenesis.

### 3.4 High-throughput sequencing of TNF-α+IL-1β-Exos and Con-Exos

**Figure 4:**
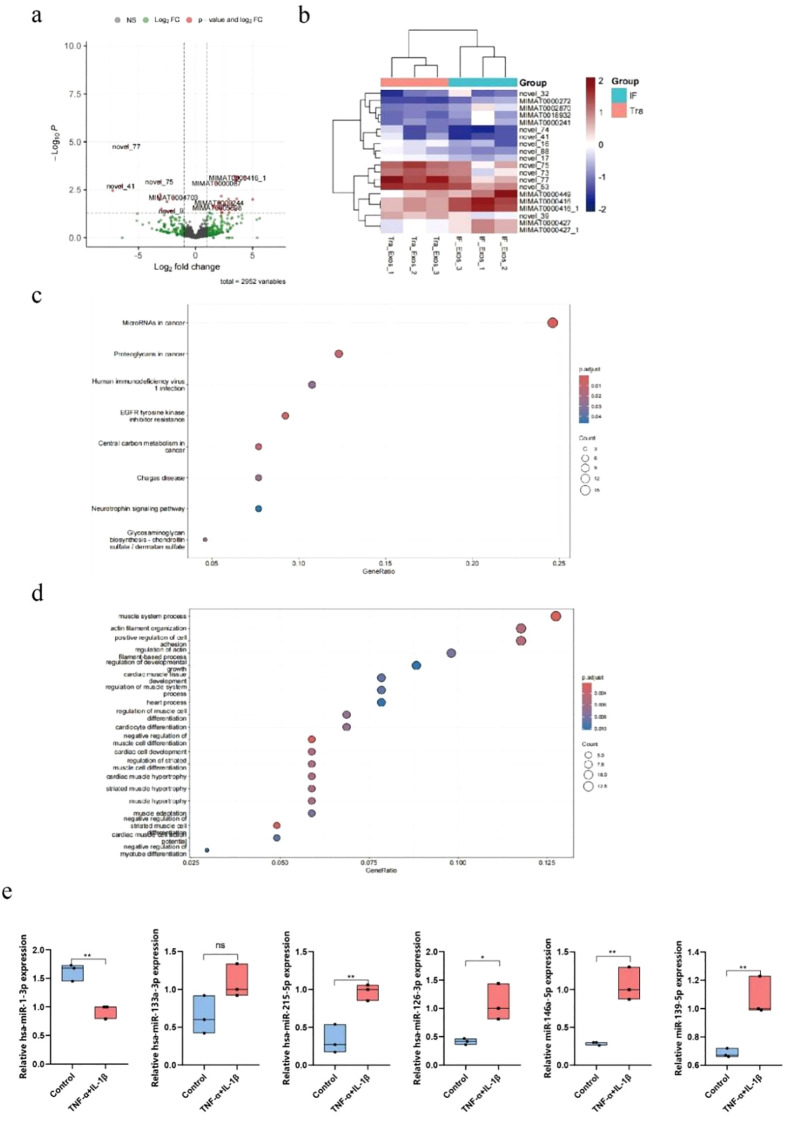
High-throughput sequencing of TNF-α+IL-1β-Exos and Con-Exos. **(a)** Volcano plot of differentially expressed miRNAs, red indicates upregulated genes and green indicates downregulated genes. **(b)** Heatmap of differentially expressed gene clustering. **(c)** GO enrichment analysis of differentially expressed genes. **(d)** KEGG pathway analysis of differentially expressed genes. **(e)** qPCR validation results of differentially expressed genes (*P<0.05, **P<0.01).

To investigate the potential mechanisms of TNF-α+IL-1β-Exos and Con-Exos in wound healing, we extracted and performed high-throughput sequencing of miRNAs from both groups of Exos. The results revealed that compared to the control group, the expression levels of miR-1-3p, miR-133a-3p, miR-215-5p, miR-126-3p, miR-146a-5p, and miR-139-5p were significantly upregulated in the TNF-α+IL-1β-Exos group (**Figures 4A, B**). To further validate the differential expression of these miRNAs, qPCR was performed using U6 as the internal reference gene. U6 snRNA, a highly abundant cellular RNA with remarkably stable expression levels across different cell types, tissues, and experimental conditions, represents an ideal internal control due to its excellent amplification efficiency and superior detection sensitivity (15). The results confirmed that the increased expression of miR-215-5p, miR-126-3p, miR-146a-5p, and miR-139-5p was statistically significant (**Figure 4E**).

KEGG and GO functional enrichment analyses of the sequencing data, conducted using TargetScan and miRanda databases, demonstrated that the upregulated miRNAs in TNF-α+IL-1β-Exos were predominantly associated with the regulation of muscle formation and angiogenesis (**Figures 4C, D**). Among these, miR-215-5p played a particularly prominent role in vascular regulation. Given its significant impact, we selected miR-215-5p as the target miRNA for further in-depth investigation.

### 3.5 TNF-α+IL-1β-Exos promote upregulation of miR-215-5p and *WNK1* in HUVECs

**Figure 5:**
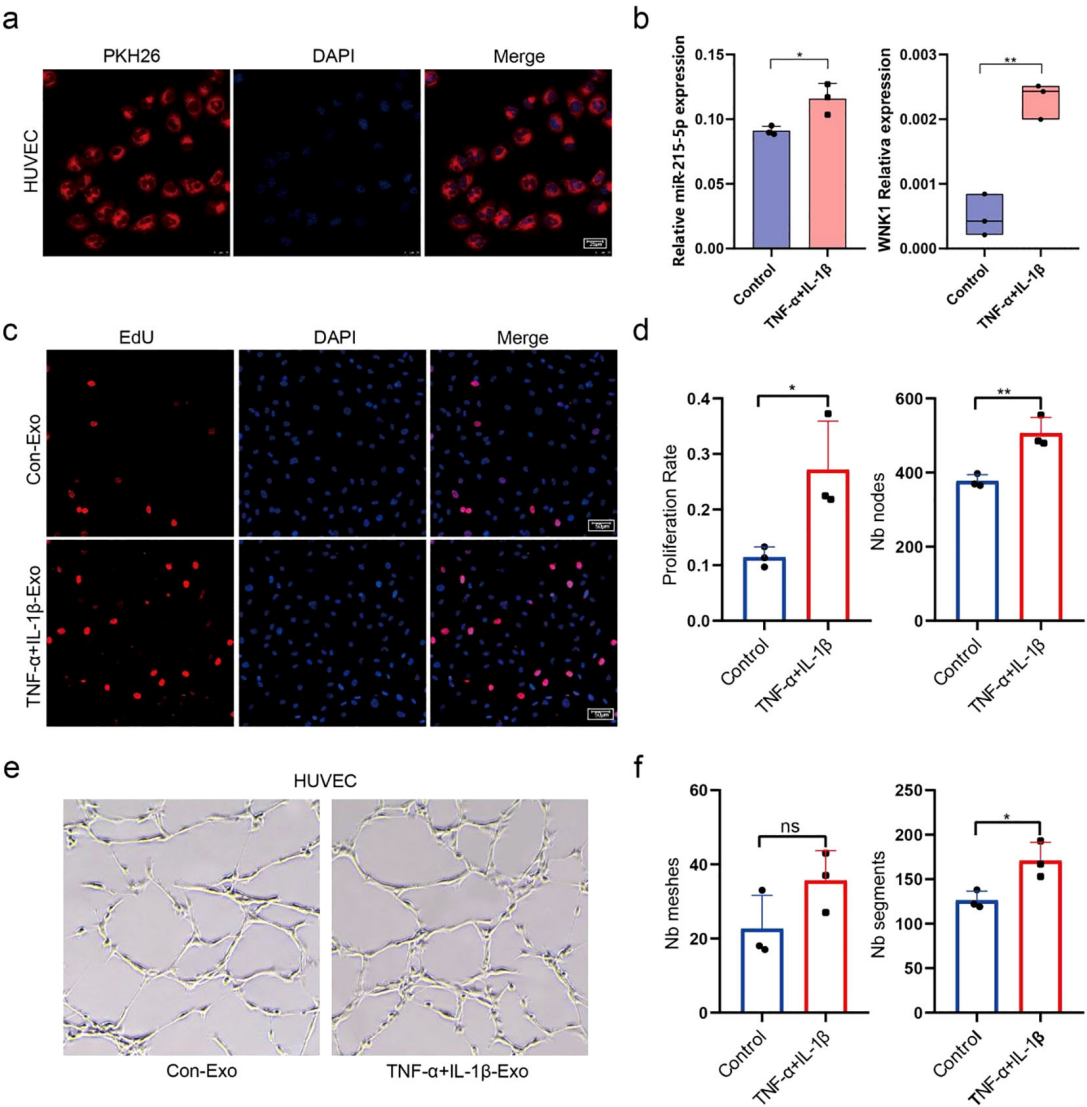
TNF-α+IL-1β-Exos promote upregulation of miR-215-5p and *WNK1* in HUVECs. **(a)** Uptake of PKH26-labeled Exos by HUVECs (800×, scale bar = 25 µm). **(b)** qPCR analysis of miR-215-5p and *WNK1* expression in HUVECs. **(c)** EdU proliferation assay in HUVECs (400×, scale bar = 50 µm). **(d)** Quantification of EdU assay (left) and tube formation Node count (right). **(e)** Tube formation assay of HUVECs (200×, scale bar = 100 µm). **(f)** Statistical data of vascular mesh area and segment number (*P < 0.05, **P < 0.01, n = 3).

Fluorescence microscopy confirmed successful uptake of PKH26-labeled Exos by HUVECs after 48 h of co-culture (**Figure 5A**). To further examine the effects of Exos on HUVECs, we conducted qPCR analysis on cells that had internalized either TNF-α+IL-1β-Exos or Con-Exos. The results showed that TNF-α+IL-1β-Exos treatment significantly upregulated the expression of the target gene miR-215-5p and its downstream effector *WNK1* compared to the control group (**Figure 5B**). Moreover, EdU assays revealed that TNF-α+IL-1β-Exos markedly enhanced HUVECs proliferation (**Figures 5C, D** left). Additionally, tube formation assays demonstrated that TNF-α+IL-1β-Exos significantly increased the number of nodes, meshes, and vascular segments in HUVECs networks, further supporting their pro-angiogenic effects (**Figures 5D–F** right).

### 3.6 MiR-215-5p promotes high expression of the *WNK1/p-Smad3/VEGF-A in vitro*

**Figure 6:**
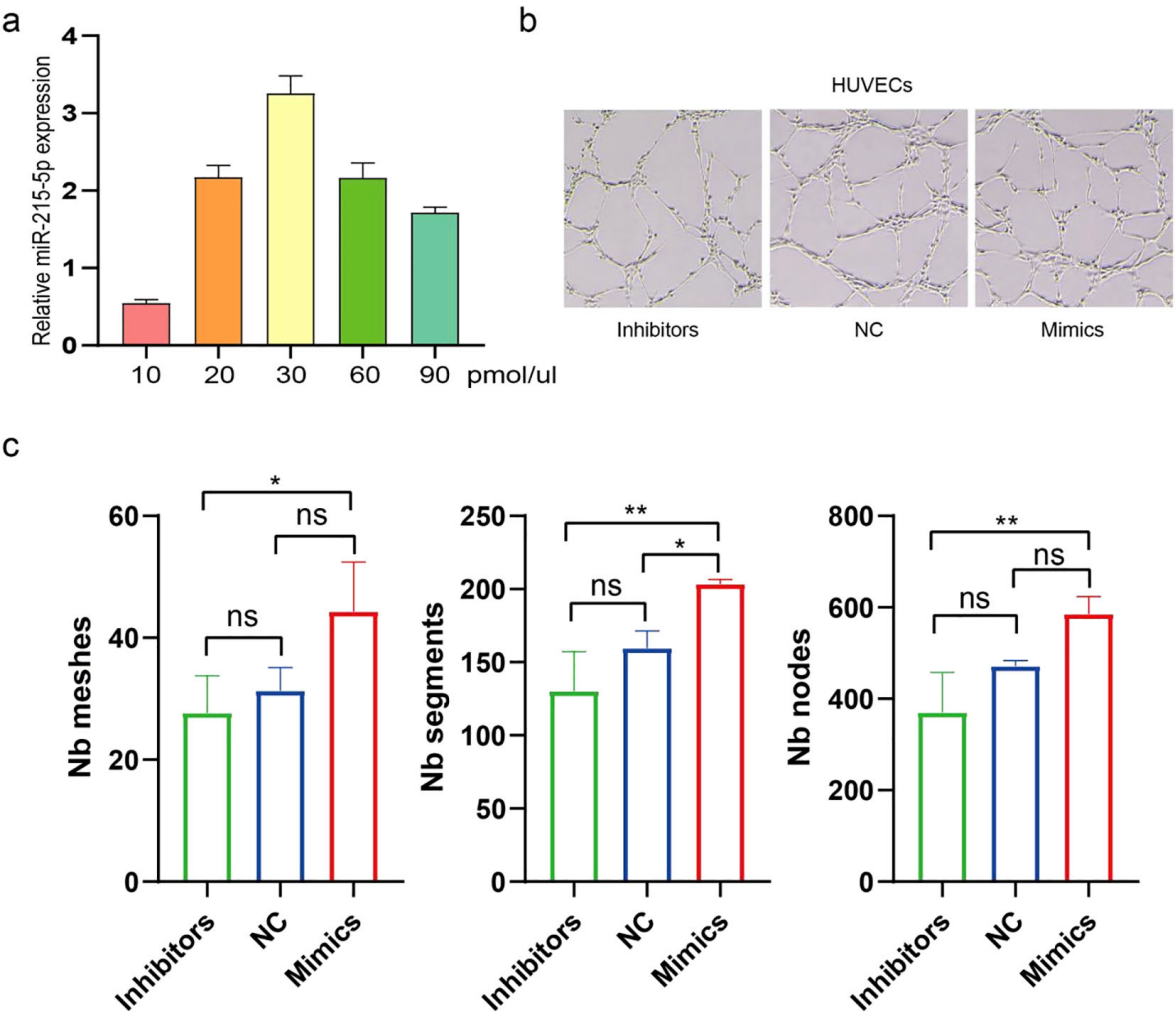
MiR-215-5p promote high expression of the *WNK1*/*p-Smad3*/*VEGF-A in vitro*. **(a)** Transfection efficiency of miR-215-5p mimics. **(b)** Tube formation assays in HUVECs transfected with miR-215-5p mimics, miR-215-5p inhibitor, and NC (200×, scale bar = 100 µm). **(c)** Quantitative analysis of Node, Mesh, and Segment counts in HUVECs (*P < 0.05, **P < 0.01, n = 3).

**Figure 7:**
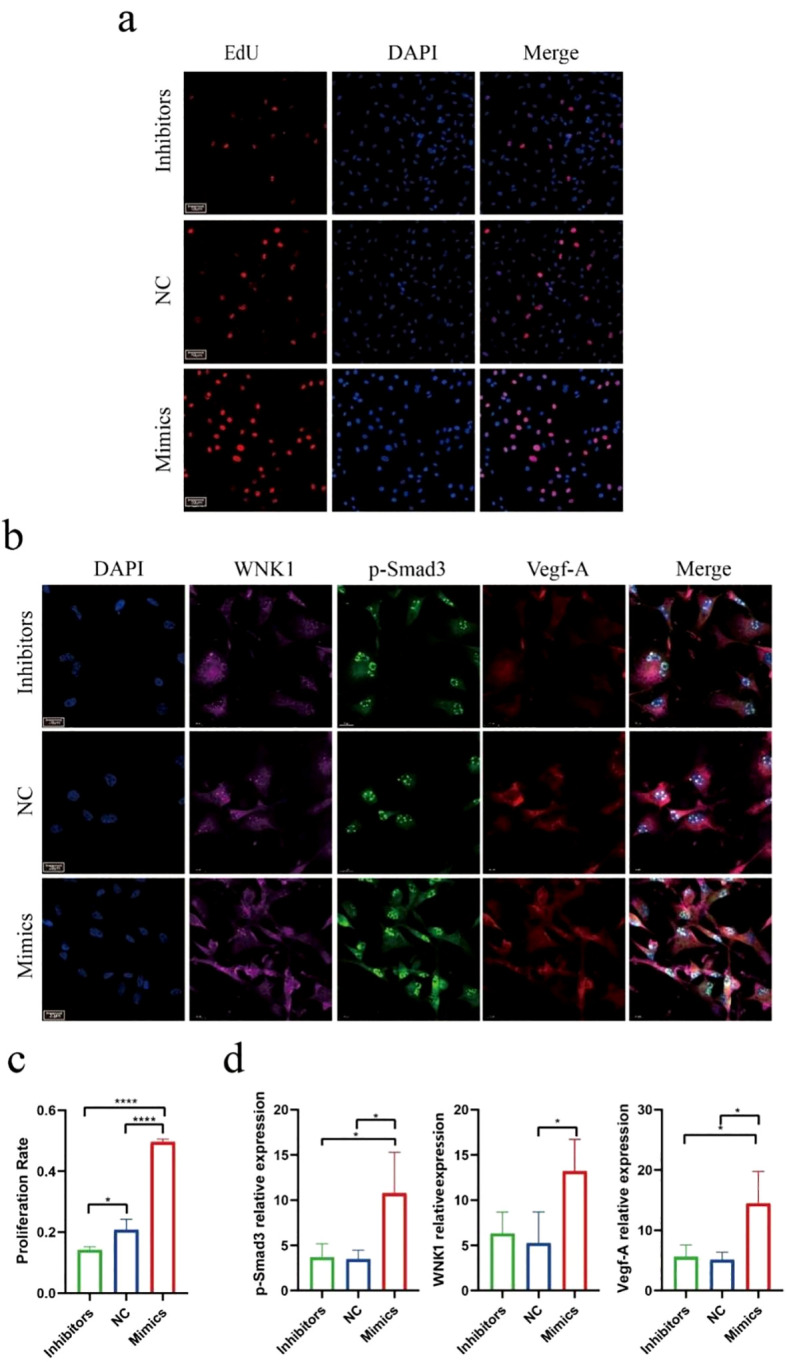
MiR-215-5p promote high expression of the *WNK1*/*p-Smad3*/*VEGF-A in vitro*. **(a)** EdU assay imaging of miR-215-5p mimics and inhibitors (400×, scale bar = 50 µm). **(b)** Immunofluorescence staining of the downstream pathway components *WNK1*, *p-Smad3*, and *VEGF-A* following transfection with miR-215-5p mimics, inhibitors and NC (1000×, scale bar = 20 µm. **(c)** Quantitative analysis of EdU assay. **(d)** Statistical results of relative fluorescence intensity of *WNK1*, *p-Smad3*, and *VEGF-A* (*P < 0.05, ****P < 0.0001, n = 3).

To investigate the functional role of miR-215-5p in HUVECs, the cells were transfected with miR-215-5p mimics, inhibitor, and PBS as a negative control (NC). The optimal transfection efficiency was achieved at a concentration of 30 pmol/μL (**Figure 6A**). Subsequent functional assays revealed that miR-215-5p mimics significantly enhanced the tube-forming capacity of HUVECs, whereas the inhibitors showed no significant inhibitory effect compared to the NC group (**Figures 6B, C**).EdU assays demonstrated that miR-215-5p mimics promoted HUVECs proliferation (**Figures 7A, C**). Furthermore, immunofluorescence analysis of downstream targets showed that miR-215-5p mimics markedly upregulated the expression of *WNK1*, *p-Smad3*, and *VEGF-A* (**Figures 7B, D**). In contrast, neither the NC nor the miR-215-5p inhibitor group exhibited significant changes in these target genes. These findings suggest that under basal conditions, miR-215-5p is expressed at relatively low levels in HUVECs, which may account for the lack of phenotypic effects upon its inhibition.

### 3.7 MiR-215-5p promotes wound healing *in vivo*

**Figure 8:**
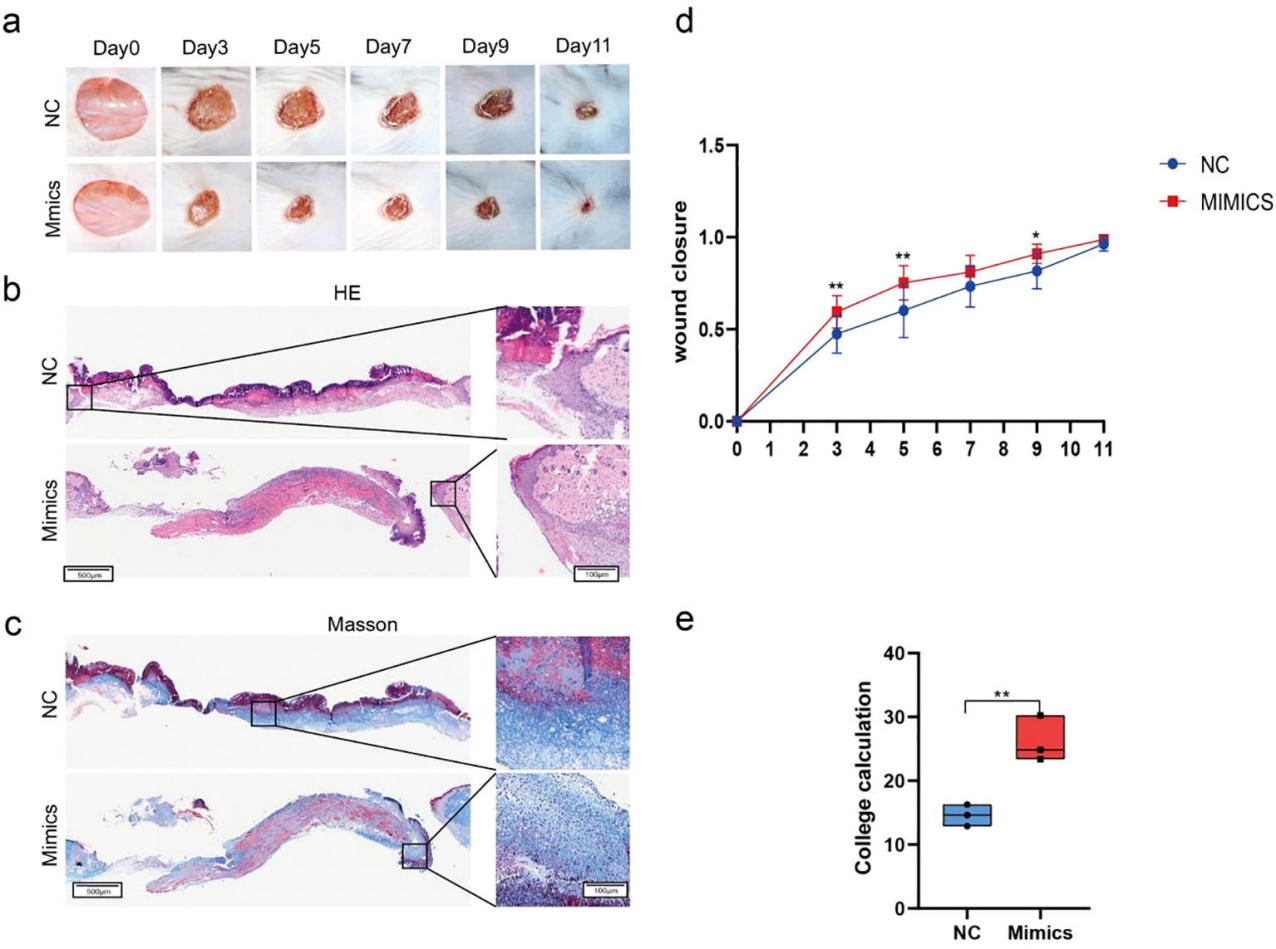
MiR-215-5p promotes wound healing *in vivo*. **(a)** Representative images of wound healing in mice (Scale bar = 1 cm, n = 7). **(b)** H&E staining images of wounds in mice on day 5 (200×, Scale bar = 100 µm). **(c)** Masson’s trichrome staining images of wounds in mice on day 5 (200×, Scale bar = 100 µm). **(d)** Quantitative analysis of wound healing rates at different stages. **(e)** Quantification of collagen deposition based on Masson’s trichrome staining (*P < 0.05, **P < 0.01, n = 3).

Through our experiments, we confirmed that miR-215-5p mimics significantly upregulate the expression of the target genes *WNK1*, *p-Smad3*, and *VEGF-A in vitro*, whereas no significant regulatory effects were observed in the NC group or the miR-215-5p inhibitors group. Based on these findings, we next investigated the impact of miR-215-5p mimics on wound healing in mice.We created full-thickness circular wounds (1 cm diameter) on the dorsal skin of mice and monitored healing progression. Photographic analysis revealed significantly accelerated wound closure in the miR-215-5p mimics group on days 3, 5, and 9 compared to controls (**Figures 8A, D**). Histopathological evaluation further supported these observations: HE staining demonstrated enhanced re-epithelialization (**Figure 8B**), while Masson’s trichrome staining showed increased collagen deposition with a more organized fiber arrangement in the mimics-treated group (**Figures 8C, E**).

**Figure 9:**
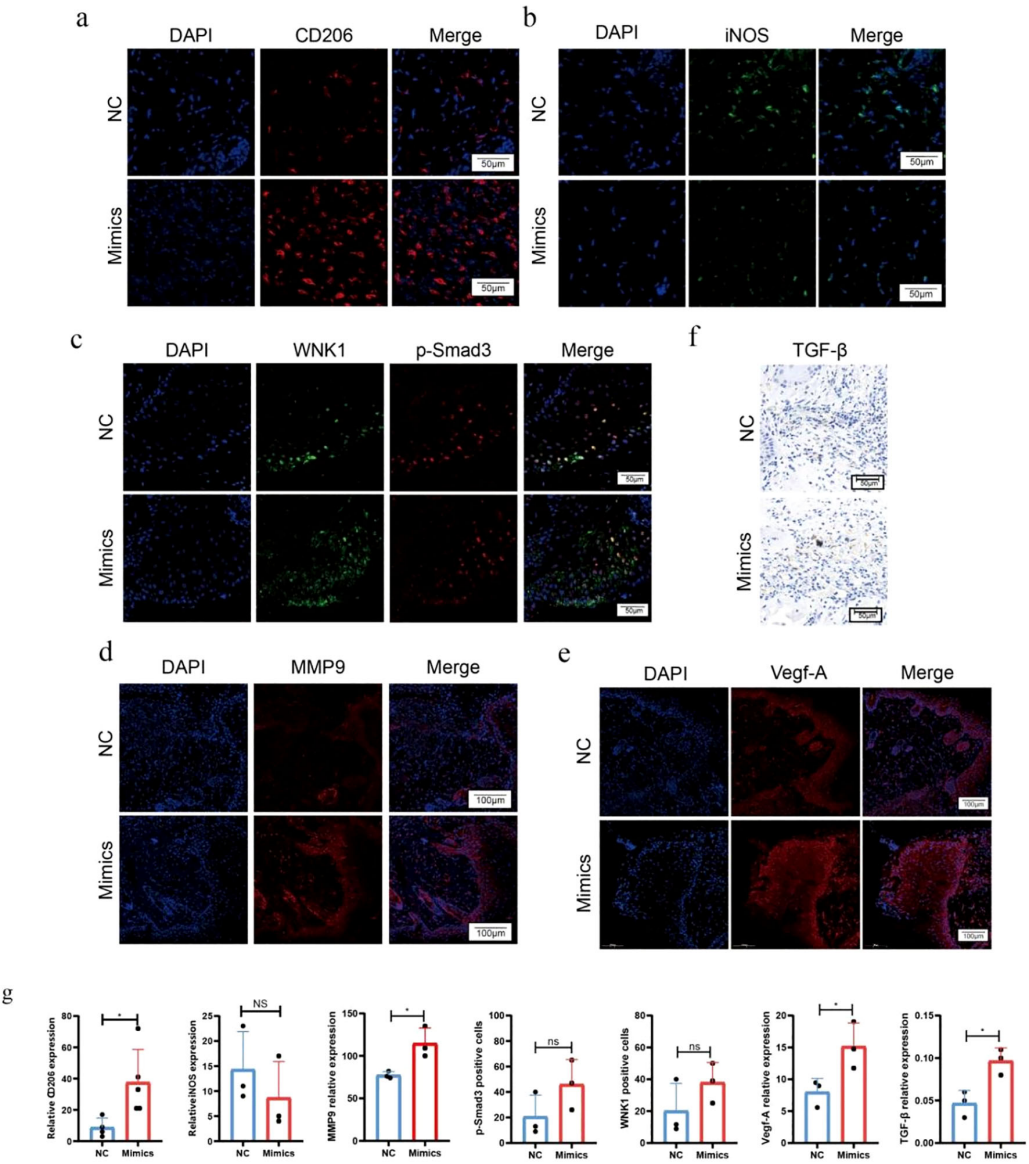
MiR-215-5p promotes wound healing *in vivo*. **(a)** Immunofluorescence staining images for CD206 in wound tissue from mice (400×, scale bar = 50 µm). **(b)** Immunofluorescence staining images for iNOS in wound tissue from mice (400×, scale bar = 50 µm). **(c)** Immunofluorescence staining images for *WNK1*/*p-Smad3* in wound tissue from mice (400×, scale bar = 50 µm). **(d)** Immunofluorescence staining images for *MMP9* in wound tissue from mice (200×, scale bar = 100 µm). **(e)** Immunofluorescence staining images for *VEGF-A* in wound tissue from mice (200×, scale bar = 100 µm). **(f)** Immunofluorescence staining images for TGF-β in wound tissue from mice (400×, scale bar = 50 µm). **(g)** Statistical results of immunofluorescence staining (*P < 0.05, n = 3).

To further elucidate the role of miR-215-5p mimics in wound healing, we employed immunofluorescence to analyze key cellular changes during the healing process. Macrophages, which play a pivotal role in wound repair are broadly classified into pro-inflammatory M1 and anti-inflammatory M2 subtypes. In the early phases of healing, M1 macrophages dominate, promoting an inflammatory microenvironment. As healing progresses, these cells gradually transition to the M2 phenotype, which suppresses inflammation and facilitates tissue repair (16).Using CD206 and iNOS as specific markers for M2 and M1 macrophages, respectively, our immunofluorescence staining revealed that miR-215-5p mimic treatment significantly increased the number of CD206^+^ cells (**Figure 9A**) while reducing iNOS expression (**Figure 9B**). These findings align with our *in vitro* transfection results in HUVECs, further supporting miR-215-5p’s role in modulating macrophage polarization. Additionally, we investigated the underlying molecular mechanisms. The expression of *WNK1*, a direct target of miR-215-5p, was elevated, leading to increased *p-Smad3* (**Figure 9C**). This activation subsequently upregulated *MMP9* (**Figure 9D**), a key mediator of extracellular matrix remodeling, and also upregulates the expression of VEGF-A (**Figure 9E**). Furthermore, we detected the expression of TGF-β, a ligand of the HUVECs envelope and an upstream signaling molecule of *p-Smad3*, which showed an increasing trend compared to the control group Under the influence of miR-215-5p mimics (**Figure 9F**). Statistical data for each experiment are presented in **Figure 9G**.

## 4 Discussion

In this study, we demonstrated that exosomes derived from hUC-MSCs preconditioned with TNF-α and IL-1β exhibited enhanced therapeutic efficacy in wound healing as compared to Con-Exos. The dual cytokine pretreatment strategy effectively mimicked the inflammatory microenvironment of early wound healing and enabled hUC-MSCs to produce exosomes with specific functions. TNF-α, a key early inflammatory cytokine that activates the *NF-κB* pathway to stimulate endothelial cell proliferation, migration, and tubulointerstitial structure formation, can also indirectly, by regulating the expression of other growth factors (e.g.*VEGF-A*), and plays a dual role in the wound healing process: on the one hand, it effectively removes pathogens and necrotic tissues by chemotaxis of inflammatory cells, such as neutrophils and macrophages, and enhances their phagocytic activity; on the other hand, the activated inflammatory cells release a variety of pro-angiogenic factors, thus closely correlating inflammatory response with the angiogenic process (17, 18). Besides, IL-1β promotes fibroblast proliferation and extracellular matrix (ECM) remodeling, providing the necessary structural support for angiogenesis (19). These theoretical evidences provide ideas and methods to study the role of Exos in inflammation-related wound healing.

High-throughput sequencing revealed significant upregulation of miR-215-5p, miR-126-3p, and miR-146a-5p in TNF-α+IL-1β-Exos, with miR-215-5p emerging as the core regulator. Bioinformatics analysis indicated that these miRNAs primarily target *WNK1* (20), *VCAM1* (21), *PIK3R2*, *VEGF-A*, *CXCR4*, *IRAK1* (22), and *METTL7A*, collectively participating in pathways related to vascular endothelial cell adhesion and angiogenesis, which may underlie the wound-healing enhancement by cytokine-stimulated Exos. TargetScan and miRanda database analyses suggested that miR-215-5p potentially regulates *WNK1* expression. Existing studies identify *WNK1* as a potential oncogenic target that promotes epithelial-mesenchymal transition (EMT) in tumor cells and endothelial-mesenchymal transition (EndMT) and angiogenesis in endothelial cells (20). These biological processes are critical in wound healing (23, 24), implicating *WNK1* as a key signaling molecule in this pathway. Subsequent *in vitro* and *in vivo* studies employing cell transfection techniques and murine wound models elucidated the mechanistic pathway through which miR-215-5p promotes wound healing. *In vitro* functional validation demonstrated that miR-215-5p mimics significantly enhance HUVECs proliferation and tubular structure formation by activating the *WNK1*/*p-Smad3*/*VEGF-A* signaling axis. Notably, miR-215-5p inhibitors exhibited minimal suppressive effects, suggesting low baseline levels of miR-215-5p in HUVECs and highlighting its therapeutic potential through overexpression. *In vivo* experiments confirmed that miR-215-5p mimics accelerated wound closure, increased collagen deposition, and promoted macrophage polarization from M1 to M2 phenotype, further underscoring this miRNA’s pivotal role in angiogenesis and tissue repair. Investigation into the mechanism of miR-215-5p-mediated neovascularization revealed its regulation of endothelial cell proliferation, migration, and differentiation through targeted genes, thereby driving vasculogenesis. Wound healing is a multi-stage collaborative process, after the initial inflammatory phase eliminates pathogenic factors, nascent vascular networks provide oxygen and nutrients for tissue repair, while fibroblast-mediated ECM remodeling and immune cell-regulated microenvironments jointly propel regeneration (25). As a key regulatory molecule, miR-215-5p plays a central role in angiogenesis but requires networked interactions with multiple cell types (endothelial cells, fibroblasts, macrophages, etc.) and pathways (e.g.*VEGF*, *TGF-β*) to ultimately achieve wound repair.

Despite these promising findings, several limitations warrant consideration. First, the purity and yield of Exos isolated via ultracentrifugation may impact their functional potency (26), as impurities or degradation could compromise therapeutic efficacy. Future studies may benefit from combining ultracentrifugation with size-exclusion chromatography or immunomagnetic separation to enhance Exos purity (15). Moreover, engineering Exos to improve their stability, targeting specificity, and cargo capacity could optimize their clinical applicability (27). Second, while murine models are informative, they may not fully recapitulate the dynamics of human wound healing (28). Subsequent research should prioritize porcine skin defect models or diabetic wound models that more closely approximate human pathophysiology to enhance translational relevance. Follow-up studies could employ siRNA-mediated *WNK1* or *Smad3* knockdown to delineate their specific contributions to wound healing in cellular or animal models. When investigating miR-215-5p functionality, its multi-target regulatory network must be holistically considered while controlling for potential confounding factors such as other target genes like *XIAP* (29) and *ZEB2*. *XIAP*, an apoptosis inhibitory protein, may influence wound healing by modulating cellular survival-apoptosis balance, whereas *ZEB2*, a key EMT regulator, not only drives tumor metastasis but also participates in angiogenesis and hepatocellular carcinoma recurrence (30). Through targeting these genes, miR-215-5p may exert pleiotropic regulatory effects across biological processes, underscoring the need for integrative multi-target mechanistic studies to fully elucidate its regulatory network and therapeutic potential in wound healing.

In summary, our study elucidates a novel mechanism by which TNF-α+IL-1β preconditioned hUCMSCs-Exos accelerate wound healing through miR-215-5p-mediated activation of the *WNK1*/*p-Smad3*/*VEGF-A* axis. These findings not only deepen our understanding of Exos-based regenerative therapies but also highlight the potential of cytokine priming to enhance Exos functionality. Future research should focus on optimizing Exos isolation techniques, validating mechanisms in clinically relevant models, and exploring combinatorial strategies to maximize therapeutic outcomes for chronic or refractory wounds.

## 5 Conclusion

Our study established that TNF-α+IL-1β preconditioning endows hUCMSCs-Exos with superior wound-healing capabilities, primarily through the enrichment of miR-215-5p. This miRNA orchestrates tissue repair by activating the *WNK1*/*p-Smad3*/*VEGF-A* axis, driving endothelial cell proliferation and angiogenesis. We also demonstrated that the expression of miR-215-5p was extremely low in untreated tissues, its endogenous levels in unstimulated cells were insufficient to influence repair processes. These findings underscore the promise of cytokine-primed exosomes and miRNA-based therapies in regenerative medicine. However, advancing this technology to the clinic will require rigorous characterization of Exos preparations, validation in physiologically relevant models, and exploration of combinatorial strategies to enhance efficacy. Our work lays a foundation for developing novel Exos therapeutics to address unmet needs in chronic wound management.

## Data Availability

The original contributions presented in the study are included in the article/supplementary material. Further inquiries can be directed to the corresponding author.
